# Double Hopf bifurcation and chaotic dynamics in a periodically-forced SIR model

**DOI:** 10.1038/s41598-025-28016-3

**Published:** 2025-12-24

**Authors:** João P. S. Maurício de Carvalho

**Affiliations:** Research on Economics, Management and Information Technologies, Prince Henry Portucalense University, Rua Dr. António Bernardino de Almeida 541, 4200-072 Porto, Portugal

**Keywords:** SIR model, Hopf bifurcation, Seasonality, Strange attractors, Torus-breakdown, Computational biology and bioinformatics, Mathematics and computing, Physics

## Abstract

We perform a qualitative analysis of a periodically-forced SIR model which incorporates the disease transmission rate by direct contact with the natural viral source, in addition to the classic disease transmission rate between individuals. We direct this work towards two main topics: (i) in the absence of seasonality, the endemic equilibrium point (and unique) undergoes both supercritical and subcritical *Hopf* bifurcations. We identify a specific range of values for the disease transmission rate $\beta$, for which the system exhibits an attracting periodic solution while the equilibrium is unstable; (ii) in the presence of seasonality, we prove via *torus-breakdown* theory that the system exhibits *strange attractors* (observable chaos). These findings reveal that small changes in parameters can generate complex epidemic dynamics, becoming very difficult to control. All findings are derived analytically and supported by numerical simulations.

## Introduction

Compartmental mathematical models are commonly used to analyze the spread of infectious diseases, such as measles, influenza (or seasonal flu), tuberculosis, COVID-19, among others^1–3^. W. O. Kermack and A. G. McKendrick published in 1927, 1932 and 1933 a trilogy of articles to explain the spread of infectious diseases, with the first of these focused on epidemics^4^.

The SIR model, proposed by W. O. Kermack and A. G. McKendrick^5^, is one of the simplest compartmental models used to understand the spread of diseases in a population. It organizes individuals into three groups: *Susceptibles*, *Infectious* (or *Infected*) and *Recovered*, and the system of ordinary differential equations (ODE) that governs the transmission of the disease between individuals is given by^4^:

$$\begin{aligned} (\dot{S}, \, \dot{I}, \, \dot{R}) \, = \, (- \beta S I, \, \beta S I - g I, \, g I) \, , \end{aligned}$$

where $N = S + I + R$ represents the total number of individuals, $\beta$ is the disease transmission rate and *g* is the disease recovery rate.

Over time, several models inspired by the classic SIR model have emerged, including the seasonally forced SIR model. In 1976, Dietz introduced seasonality into a mathematical model for the transmission of infectious diseases for the first time^6^. Seasonality incorporates regular fluctuations influenced by factors such as human behavior, environmental changes, school schedules, climatic variations and political decisions (See^7^and references therein). Adding seasonality to epidemiological models increases the realism of the dynamics, since certain diseases have higher incidence rates during specific times of the year. In this way, if seasonality is introduced into the disease transmission rate, it can be modeled through periodic functions^8–11^.. Periodically-forced SIR models, in which seasonal variations in the disease are not neglected, are naturally more complex, with richer and more sophisticated dynamics than traditional models^12^.

In the literature there are mathematical models applied to epidemiology that focus on the analysis of bifurcations and the impact of seasonality on the dynamics of infectious diseases:

### Bifurcation analysis

Maurício de Carvalho and Rodrigues^13^ analyzed a modified SIR model with constant vaccination strategy. In the parameter space $(\mathcal {R}_0, p)$, where $\mathcal {R}_0$ is the basic reproduction number and *p* is the proportion of *Susceptible* individuals successfully vaccinated at birth, the authors analytically proved that the endemic equilibrium is a codimension two singularity – called *Double-zero* bifurcation (identical to the *Bogdanov-Takens* bifurcation) – and identified regions where the disease persists in the population. Karaji *et al.*^14^ worked on a SIR model with a nonlinear incidence rate. They conducted a stability analysis of the equilibria, concluding that the disease-free equilibrium is asymptotically stable when $\mathcal {R}_0 < 1$ and unstable otherwise. Additionally, the authors discovered that the model exhibits a *Hopf* and a transcritical bifurcations.

### Seasonality

Bilal *et al.*^15^ studied different epidemic models where they introduced seasonality in the disease transmission rate through non-autonomous periodic functions. The authors concluded that the emergence of non-chaotic *strange attractors* could predict epidemic outbreaks. Keeling *et al.*^12^ explored the impact of seasonality on a modified SIR model, focusing on diseases such as measles, whooping cough and rubella. Their results revealed that predominantly childhood illnesses, which are subject to seasonal variations, have a more complex dynamic than initially thought. Maurício de Carvalho and Rodrigues^16^ studied a modified SIR model with seasonality. The authors concluded that when the flow is subjected to intense seasonality, the dynamics exhibit unpredictable and chaotic behaviors (*strange attractors*).

### Novelty, goals and achievements

The proposed model introduces a direct transmission term representing infection through a natural viral source, in contrast to classical SIR models where transmission occurs only through contact with *Infectious* individuals. In addition, the transmission rate $\beta _\gamma (t)$ is periodically forced to capture seasonality. This formulation gives rise to rich and complex dynamics that deserve detailed analysis. The model is examined both with and without seasonality in the disease transmission rate through a system of ODEs. The main goal of this study is the rigorous proof of the following statements:

1. In the absence of seasonality:
  - the equilibrium point of the model undergoes a supercritical and a subcritical *Hopf* bifurcations;
  - we identify a subset of values for the disease transmission rate for which the system exhibits an attracting periodic solution and the endemic equilibrium is unstable.
2. In the presence of seasonality, the system reveals unpredictable dynamics for the flow, exhibiting abundant *strange attractors* (observable chaos) via *Torus-breakdown* theory.

All our results were obtained analytically and corroborated by numerical simulations.

### Structure

In Section “Model” we describe the model we propose and present our hypotheses and motivations for designing it. In Section "Absense of seasonality: First main result" we define a compact set for the flow of the system in absense of seasonality. Also in this section we present the first main result of this work for the model without seasonality. In Section "Seasonality and chaotic dynamics: Second main result" we present our second main result. Throughout this paper we present illustrations and numerical simulations that corroborate our analytical proofs. Section "Conclusion and discussion" provides a discussion of the work and a brief summary of comparisons with other papers in the literature.

## Model

**Fig. 1:**
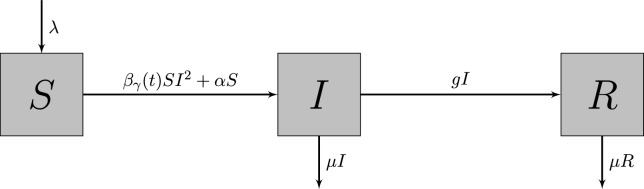
Illustration of the interactions between populations in model (1). Boxes represent the compartments of *S*, *I*, and *R*. Arrows indicate the flow between the compartments.

Using the classic SIR model^5^, we organize the population into three classes of individuals: *Susceptibles*, *Infected*/*Infectious* and *Recovered* (*R*). The variables *S*(*t*), *I*(*t*) and *R*(*t*) represent the number of individuals at time *t* in each compartment – *Susceptible*, *Infected* or *Infectious* and *Recovered*, respectively. We assume that *Susceptible* individuals have never been in contact with the disease. However, when they are in contact with the disease, either through contact with *Infectious* individuals or directly with the natural viral source, they become infected (and infectious). The *Infectious* individuals move into the *Recovered* class if they are cured and ensure immunity for life. The model we propose consists of the following non-linear system of ODE in the variables *S*, *I* and *R*, which vary in time $t\in \mathbb {R}_0^+$:

1 $$\begin{aligned} \begin{array}{lcl} \dot{X} = \mathcal {F}_\gamma (X) \quad \Leftrightarrow \quad {\left\{ \begin{array}{ll} & \dot{S} = \lambda - \beta _{\gamma } S I^2 - \left( \alpha + \mu \right) S \\ \\ & \dot{I} =\beta _{\gamma }SI^2 + \alpha S - \left( g+ \mu \right) I \\ \\ & \dot{R} = gI - \mu R \end{array}\right. } \end{array} \end{aligned}$$

where

$$\begin{array}{rcl} X(t) & =& \left( S(t), I(t), R(t) \right) , \\ \\ X(t_0) & :=& X_0 \,\,\, \text {is the initial condition}, \\ \\ \dot{X} & =& \left( \dot{S}, \dot{I}, \dot{R}\right) \,\,\, = \,\,\, \displaystyle \left( \frac{\textrm{d}S}{\textrm{d}t},\frac{\textrm{d}I}{\textrm{d}t},\frac{\textrm{d}R}{\textrm{d}t}\right) , \end{array}$$

and

2 $$\begin{aligned} \begin{array}{lcl} \beta _\gamma (t) = \beta \left( 1+\gamma \Psi (\omega t)\right) \end{array} \end{aligned}$$

is the term that governs the disease transmission rate. When $\gamma> 0$, then the disease transmission rate does not neglect seasonality. The dynamics of (1) are illustrated in Fig. 1 and we can describe the parameters of (1) as:

- λ: linear growth of *Susceptible* individuals;
- γ: seasonal variation amplitude (“measures the deformation” of the transmission rate forced by the seasonality);
- $\Psi (\omega t)$: effects of periodic seasonality over the time (with frequency $\omega> 0$);
- $\beta$: disease transmission rate, via contact with *Infectious* individuals, in the absence of seasonality ($\gamma =0$);
- $\alpha$: disease transmission rate by direct contact with the natural viral source;
- *g*: cure rate;
- $$\mu$$: death rate.

The vector field associated to (1) will be denoted by $\mathcal {F}_{\gamma }$ and its flow is $\varphi \left( t, (S_0, I_0, R_0) \right)$, $t \in \mathbb {R}_0^+$ and $(S_0, I_0, R_0) \in (\mathbb {R}_0^+)^3$.

### Compact set and invariant flow for $\mathcal {F}_{\gamma }$

The following Lemma defines a closed and bounded set 

$\mathcal {M}$ for the flow $\mathcal {F}_{\gamma }$ of the system (1) for $\gamma = 0$:

#### Lemma 1

The region defined by

$$\mathcal {M} = \left\{ (S,I,R) \in (\mathbb {R}_0^+)^3: \quad 0 \le S+I+R \le \dfrac{\lambda }{\delta }, \quad S, I, R\ge 0 \right\}$$

is positively flow-invariant for (1).

#### Proof

We can easily check that $(\mathbb {R}_0^+)^3$ is *flow-invariant*:

$$\begin{aligned} \begin{array}{lcl} \dot{S} \,\big |_{S = 0} & =& \lambda \,\,> \,\, 0 \\ \\ \dot{I} \,\big |_{I = 0} & =& \alpha S \,\,> \,\, 0 \\ \\ \dot{R} \,\big |_{R = 0} & =& g I \,\,> \,\, 0 . \end{array} \end{aligned}$$

Now, we show that $\varphi \left( t, (S_0, I_0, R_0) \right)$ is contained in $\mathcal {M}$. Let us define

$$\begin{aligned} \eta (t) = S(t) + I(t) + R(t) \ge 0 \end{aligned}$$

associated to the trajectory $\varphi \left( t, (S_0, I_0, R_0) \right)$. From (1), one knows that

$$\begin{aligned} \begin{array}{lcl} \dot{\eta } & = & \dot{S} + \dot{I} + \dot{R}\\ \\ & = & \lambda - \beta S I^2 - \alpha S -\mu S + \beta SI^2 + \alpha S - gI - \mu I + g I - \mu R\\ \\ & = & \lambda - \mu \eta . \end{array} \end{aligned}$$

Hence, we deduce that

$$\begin{aligned} \begin{array}{lcl} \dot{\eta } + \mu \eta= & \lambda . \end{array} \end{aligned}$$

Now, using the classical differential version of the Gronwall’s Lemma, we have

$$\eta (t) \le \eta _0 e^{-\mu t} - \dfrac{\lambda }{\mu } \left( e^{-\mu t}-1\right) .$$

Taking the limit when $t\rightarrow +\infty$, we get

$$\begin{aligned} 0 \,\, \le \,\, \lim _{t\rightarrow +\infty } \eta (t) \,\, \le \,\, \lim _{t\rightarrow +\infty } \left[ \eta _0 e^{-\mu t} - \dfrac{\lambda }{\mu } \left( e^{-\mu t}-1\right) \right] \,\, = \,\, \dfrac{\lambda }{\mu } \end{aligned}$$

and the result is proved. $\square$

### Hypotheses and motivation

Regarding (1), we also assume:

**(C1)**: All parameters are positive, except $\gamma$ which is non-negative;
**(C2)**: In order to simplify the notation, we denote $g + \mu$ by $\delta$ and the parameter $\alpha$ encloses disease transmission rate via contact with the natural viral source $\alpha$ and death rate $\mu$. In other terms $g + \mu = \delta$ and $\alpha + \mu \mapsto \alpha$;
**(C3)**: For $T> 0$ and $\gamma> 0$, the map $\Psi : \mathbb {R}\rightarrow \mathbb {R}^+$ is $C^3$, *T*-periodic, $\displaystyle \dfrac{1}{T} \int _0^{T} \beta _{\gamma }(t) \, \textrm{d}t> 0$ and has (at least) two nondegenerate critical points.

With respect to our model, we define ${{\Lambda } \subset \left\{ (\lambda , \beta , \alpha , \delta ) \in (\mathbb {R}^+)^4 \right\} }$ as the set of parameters. The classic SIR model of^5^, the Sel’kov system^17^ and the Dietz model^6^, where seasonal variations are considered for the first time, motivated us to develop model (1), with the following points:

- We use the linear growth (Other authors use the logistic function to model the growth of susceptible individuals, due the crowding and natural competition for resources^16,18^.) of the *Susceptibles* ($\lambda$) and the death rate of *S*, *I* and *R* ($\mu$);
- We consider the term $\beta S I^2$, that is the *Infected* influenced by the local cases^19^, instead of the classic $\beta SI$^5^;
- We assume that *Susceptible* individuals can become infected when they come into contact directly via the natural viral source ($\alpha$)^19^, instead of only becoming infected through contact with the *Infectious* ($\beta$), as is commonly considered in classical SIR models;
- The disease transmission rate ($\beta _{\gamma }$) is given by a non-autonomous periodic function (able to capture seasonal variations)^6,16^ instead of a constant map ($\beta$).

Since the first two equations of (1), $\dot{S}$ and $\dot{I}$, are independent of *R*, and making use of **(C2)**, we reduce (1) to

3 $$\begin{aligned} \begin{array}{lcl} \dot{x} = f_{\gamma }(x) \quad \Leftrightarrow \quad {\left\{ \begin{array}{ll} & \dot{S} = \lambda - \beta _{\gamma }S I^2 - \alpha S\\ \\ & \dot{I} =\beta _{\gamma }SI^2 + \alpha S - \delta I , \end{array}\right. } \end{array} \end{aligned}$$

with $$x=(S,I)\in (\mathbb {R}_0^+)^2$$.

## Absense of seasonality: first main result

In this section, we are going to analyze the system (3) without seasonality ($\gamma = 0$). Therefore, system (3) is rewritten as

4 $$\begin{aligned} {\left\{ \begin{array}{ll} & \dot{S} = \lambda - \beta S I^2 - \alpha S \\ \\ & \dot{I} =\beta SI^2 + \alpha S - \delta I . \end{array}\right. } \end{aligned}$$

### Absence of disease-free equilibria

Using **(C1)**, we obtain the equilibria of system (4). If we assume $I = 0$, from the first equation we get $\dot{S} = \lambda - \alpha S = 0$, leading to $S = \lambda / \alpha$. Substituting this into the second equation gives $\dot{I} = \lambda \ne 0$, showing that no equilibrium with $I = 0$ exists. Hence, we construct the following Lemma:

#### Lemma 2

System (4) has a single equilibrium point given by

$$\begin{aligned} P = \left( \dfrac{\delta ^2 \lambda }{\alpha \delta ^2 + \beta \lambda ^2},\dfrac{\lambda }{\delta }\right) , \end{aligned}$$

and it is endemic.

#### Proof

Solving $\dot{S} = 0$ and $\dot{I} = 0$ we get

$$\begin{aligned} & {\left\{ \begin{array}{ll} & \lambda - \beta S I^2 - \alpha S = 0\\ & \beta SI^{2} + \alpha S - \delta I = 0 \end{array}\right. } \\ \\ \Leftrightarrow & {\left\{ \begin{array}{ll} & S = \dfrac{\lambda }{\beta I^2 + \alpha } \\ & \beta \left( \dfrac{\lambda }{\beta I^2 + \alpha } \right) I^2 + \alpha \left( \dfrac{\lambda }{\beta I^2 + \alpha } \right) - \delta I = 0 \end{array}\right. } \\ \\ \Leftrightarrow & {\left\{ \begin{array}{ll} & S = \dfrac{\lambda }{\beta I^2 + \alpha } \\ & \beta \lambda I^2 + \alpha \lambda - \delta I \left( \beta I^2 + \alpha \right) = 0 \end{array}\right. }\\ \\ \Leftrightarrow & {\left\{ \begin{array}{ll} & S = \dfrac{\lambda }{\beta I^2 + \alpha }\\ & - \beta \delta I^3 + \beta \lambda I^2 - \alpha \delta I + \alpha \lambda = 0 \end{array}\right. }\\ \\ \Leftrightarrow & {\left\{ \begin{array}{ll} & S = \dfrac{\delta ^2 \lambda }{\alpha \delta ^2 + \beta \lambda ^2}\\ & I = \dfrac{\lambda }{\delta }. \end{array}\right. } \end{aligned}$$

Computing $- \beta \delta I^3 + \beta \lambda I^2 - \alpha \delta I + \alpha \lambda = 0$, we have three solutions for *I*: $I_1 = \frac{\lambda }{\delta }$, $I_2 = - \frac{\sqrt{-\alpha \beta }}{\beta }$ and $I_3 = \frac{\sqrt{-\alpha \beta }}{\beta }$. Since *I* represents a quantity (in terms of population), we ignore $I_2$ and $I_3$ solutions and substitute $I_1$ in $S = \frac{\lambda }{\beta I^2 + \alpha }$ to obtain $S = \frac{\delta ^2 \lambda }{\alpha \delta ^2 + \beta \lambda ^2}$. Therefore, system (4) has a single equilibrium point

5 $$\begin{aligned} P = \left( S, I \right) = \left( \dfrac{\delta ^2 \lambda }{\alpha \delta ^2 + \beta \lambda ^2},\dfrac{\lambda }{\delta }\right). \end{aligned}$$

$$\square$$

In fact, *P* is endemic, that is, the equilibrium point in the phase space (*S*, *I*) has a non-zero second component. Since *P* is endemic and unique, we conclude that there are no disease-free equilibria for system (4).

### First main result

Here we state the first result of this work. The subsequent Subsection reveal the proof of the first Theorem.

Before going forward, we establish the following constants to be utilized throughout this text:

$$\begin{aligned} \beta _1:= & \dfrac{\left( -2 \alpha + \delta - \sqrt{-8 \alpha \delta + \delta ^2} \right) \delta ^2}{2 \lambda ^2} \\ \\ \beta _2:= & \dfrac{\left( -2 \alpha + \delta + \sqrt{-8 \alpha \delta + \delta ^2} \right) \delta ^2}{2 \lambda ^2} \,\,. \end{aligned}$$

It is easy to conclude that $\beta _1 < \beta _2$ and $\beta _{1,2} \in \mathbb {R}^+$ if and only if $8\alpha < \delta$. If $8\alpha = \delta$, then $\beta _1 = \beta _2$.

#### Theorem 1

There is a non-empty open set $\mathcal {U}_1\subset \Lambda$ for which *P*:

1. is a stable focus for $\beta < \beta _1$ and $\beta> \beta _2$ and an unstable focus for $\beta \in (\beta _1, \beta _2)$;
2. undergoes a supercritical Hopf bifurcation at $\beta = \beta _1$;
3. undergoes a subcritical Hopf bifurcation at $\beta = \beta _2$.

#### Proposition 1

There is a non-empty open set $\mathcal {U}_2\subset \Lambda$ for which the flow of (4) exhibits an attracting periodic solution.

**Fig. 2:**
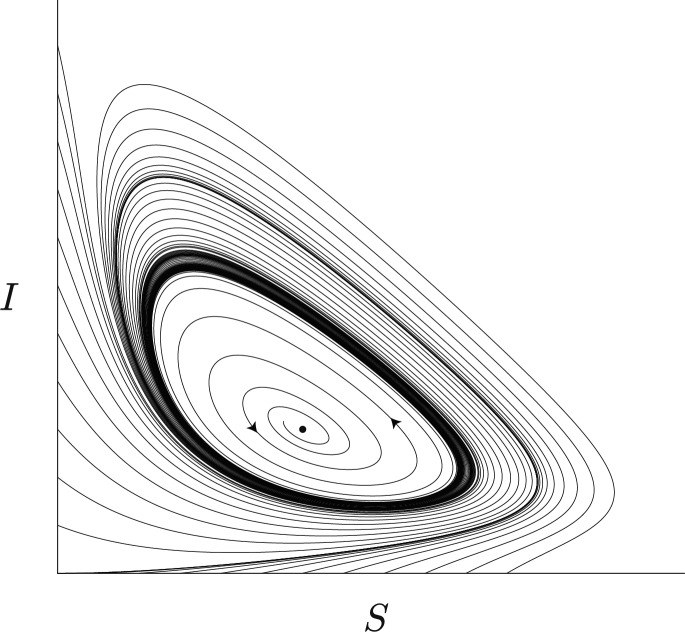
Phase portrait of system (4). Parameter values: $\lambda = 0.9$, $\beta = \left( \beta _1 + \beta _2\right) /2$, $\alpha = 0.1$ and $\delta = 1.1$. Initial conditions $\left( S_0, I_0 \right)$ have the form $(S_0, 0)$ and $(0, I_0)$. Particularly noteworthy is the existence of an attracting periodic solution $\mathcal {C}$.

An orientable stable periodic solution emerges from a supercritical *Hopf* bifurcation and vanishes through a subcritical *Hopf* bifurcation. Figure 2 provides a numerical simulation of the emergence of an attracting periodic oribit $\mathcal {C}$.

v

#### Proof of Theorem 1 and Proposition 1

In this Subsection we construct the proof of Theorem 1 and Proposition 1. First of all, let us analyze the Lyapunov stability of *P*. The jacobian matrix of the vector field (4) at $P = (S, I) \in (\mathbb {R}_0^+)^2$ is given by

6 $$\begin{aligned} \begin{array}{lcl} \mathcal {L}(P) = \left( \begin{array}{cc} -\beta I^2 - \alpha & - 2 \beta S I \\ \beta I^2 + \alpha & 2 \beta S I - \delta \end{array}\right) \end{array} . \end{aligned}$$

Let us denote by Tr $\mathcal {L}(P)$ the trace of $\mathcal {L}(P)$.

##### Lemma 3

If $\beta < \beta _1$ or $\beta> \beta _2$, then Tr $\mathcal {L}(P) < 0$. Moreover, if $\beta _1< \beta < \beta _2$, then Tr $\mathcal {L}(P)> 0$.

##### Proof

We analyze the sign of $\textrm{Tr}$ $\mathcal {L}(P)$:

$$\begin{aligned} \textrm{Tr} \,\, \mathcal {L}(P)= & -\beta I^2 - \alpha + 2 \beta S I - \delta \\ \\ \overset{(5)}{=} & \dfrac{-\alpha \delta ^2 - \beta \lambda ^2}{\delta ^2} + \dfrac{\delta \left( -\alpha \delta ^2 + \beta \lambda ^2 \right) }{\alpha \delta ^2 + \beta \lambda ^2}\\ \\ = & \dfrac{-\left( \alpha \delta ^2 + \beta \lambda ^2 \right) \left( \alpha \delta ^2 + \beta \lambda ^2 \right) + \delta ^3 \left( -\alpha \delta ^2 + \beta \lambda ^2 \right) }{\delta ^2 \left( \alpha \delta ^2 + \beta \lambda ^2 \right) }. \end{aligned}$$

As $\delta ^2 \left( \alpha \delta ^2 + \beta \lambda ^2 \right)> 0$, if $-\left( \alpha \delta ^2 + \beta \lambda ^2 \right) \left( \alpha \delta ^2 + \beta \lambda ^2 \right) + \delta ^3 \left( -\alpha \delta ^2 + \beta \lambda ^2 \right) < 0$, then $\textrm{Tr} \,\, \mathcal {L}(P) < 0$:

7 $$\begin{aligned} & -\left( \alpha \delta ^2 + \beta \lambda ^2 \right) \left( \alpha \delta ^2 + \beta \lambda ^2 \right) + \delta ^3 \left( -\alpha \delta ^2 + \beta \lambda ^2 \right)< 0\nonumber \\ \\& \Leftrightarrow - \alpha ^2 \delta ^4 - 2 \alpha \delta ^2 \lambda ^2 \beta - \lambda ^4 \beta ^2 - \alpha \delta ^5 + \delta ^3 \lambda ^2 \beta< 0\nonumber \\ \\  & \Leftrightarrow - \lambda \beta ^2 - 2 \alpha \delta ^2 \lambda ^2 \beta + \delta ^3 \lambda ^2 \beta - \alpha \delta ^4 \left( \alpha + \delta \right)< 0 \nonumber \\ \\ & \Leftrightarrow - \lambda \beta ^2 - \lambda ^2 \left( 2\alpha - \delta \right) \delta ^2 \beta - \alpha \delta ^4 \left( \alpha + \delta \right) < 0. \end{aligned}$$

One knows that (7) is a quadratic polynomial in $\beta$ with two roots:

$$\beta _1 = \dfrac{\left( -2 \alpha + \delta - \sqrt{-8 \alpha \delta + \delta ^2} \right) \delta ^2}{2 \lambda ^2}$$ and $$\beta _2 = \dfrac{\left( -2 \alpha + \delta + \sqrt{-8 \alpha \delta + \delta ^2} \right) \delta ^2}{2 \lambda ^2}$$.

Given expression (7), we conclude that when $\beta < \beta _1$ or $\beta> \beta _2$, then $\textrm{Tr}$ $\mathcal {L}(P) < 0$. Otherwise, if $\beta \in (\beta _1, \beta _2)$, then $\textrm{Tr}$ $\mathcal {L}(P)> 0$, and the result is proved. $\square$

**Fig. 3:**
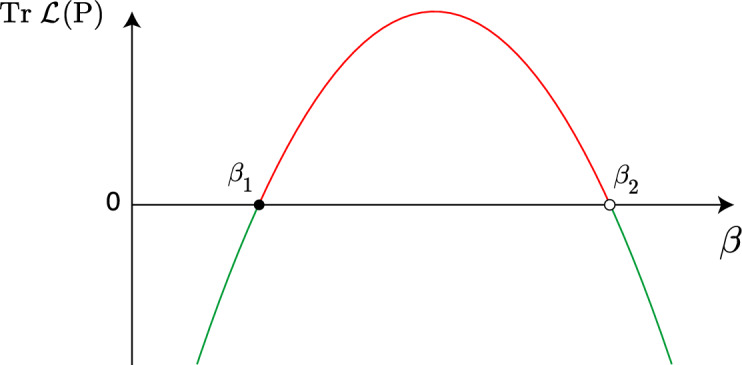
Illustration of (7). Green line: $\beta < \beta _1$ or $\beta> \beta _2 \, \Leftrightarrow \, \textrm{Tr} \,\,\mathcal {L}(P) < 0$ and *P* is stable. Red line: $\beta _1< \beta < \beta _2 \, \Leftrightarrow \, \textrm{Tr} \,\, \mathcal {L}(P)> 0$ and *P* is unstable. At $\beta = \beta _1$, *P* undergoes a supercritical *Hopf* bifurcation. At $\beta = \beta _2$, *P* undergoes a subcritical *Hopf* bifurcation.

**Fig. 4:**
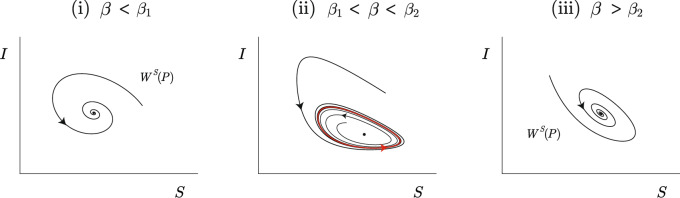
Numerical simulation of the phase diagram of (4) for different values of $\beta$. **(i)** $\beta < \beta _1$: *P* is a stable focus. **(ii)** $\beta \in (\beta _1, \beta _2)$: *P* is an unstable focus and there exists an attracting limit cycle $\mathcal {C}$. **(iii)** $\beta> \beta _2$: *P* is a stable focus. Parameter values: $\lambda = 0.9$, $\alpha = 0.1$ and $\delta = 1.1$. **(i)** $\beta = \beta _1 - 0.1$. **(ii)** $\beta = \left( \beta _1 + \beta _2 \right) /2$. **(iii)** $\beta = \beta _2 + 0.1$.

The stability of *P* is determined by the sign of the real part of the eigenvalues of $\mathcal {L}(P)$, which is equivalent to the sign of the trace of $\mathcal {L}(P)$. Through the illustration in Fig. 3, one can observe the fluctuation in the sign of $\textrm{Tr}\,\,\mathcal {L}(P)$. Instead of analytically verifying the existence of eigenvalues with an imaginary part, we have checked numerically the behavior of the flow of (4) and conclude that *P* is a focus (See Fig. 4).

##### Lemma 4

If $\beta = \beta _1$ or $\beta = \beta _2$, then *P* undergoes a Hopf bifurcation.

##### Proof

The *Hopf* bifurcation exists when the following conditions hold:

1. The eigenvalues of the map $\mathcal {L}(P)$ have the form $\pm \,i \omega$ $(\omega> 0)$, which implies that $\textrm{Tr}$ $\mathcal {L}(P) = 0$. Therefore, from Lemma 3 we know that
  $$\begin{aligned} \textrm{Tr} \,\, \mathcal {L}(P) \, = \, 0 \quad \Leftrightarrow \quad \beta \, = \, \beta _1 \quad \vee \quad \beta \, = \, \beta _2 \,. \end{aligned}$$
2. $\dfrac{\textrm{d}}{\textrm{d} \beta } \Big (\textrm{Re} \,\, \lambda _{i}(\beta ) \Big ) \Big |_{\beta \,=\, \beta _j} \ne 0$, for $i,j \in \{1,2\}$. Using the MAPLE software, we get the following eigenvalues for (6):
  8 $$\begin{aligned} \lambda _1= & \dfrac{- \alpha ^2 \delta ^4 - 2 \alpha \beta \delta ^2 \lambda ^2 - \alpha \delta ^5 - \beta ^2 \lambda ^4 + \beta \delta ^3 \lambda ^2 -\sqrt{\Delta }}{2 \left( \alpha \delta ^2+\beta \lambda ^2 \right) \delta ^2} \,\end{aligned}$$
  9 $$\begin{aligned} \lambda _2= & \dfrac{- \alpha ^2 \delta ^4 - 2 \alpha \beta \delta ^2 \lambda ^2 - \alpha \delta ^5 - \beta ^2 \lambda ^4 + \beta \delta ^3 \lambda ^2 +\sqrt{\Delta }}{2 \left( \alpha \delta ^2+\beta \lambda ^2 \right) \delta ^2} , \end{aligned}$$
  where $\Delta < 0$ (Through numerical simulations, we verify that the equilibrium point *P* is a focus. As such, the eigenvalues of $\mathcal {L}(P)$ have an imaginary part, that is $\Delta < 0$.). From (8) and (9), we obtained
  $$\begin{aligned} \dfrac{\textrm{d}}{\textrm{d} \beta } \Big (\textrm{Re} \,\, \lambda _{1}(\beta ) \Big ) \Big |_{\beta \,=\, \beta _1}= & \dfrac{\lambda ^2 \left( \sqrt{-8\alpha \delta + \delta ^2} + 8\alpha -\delta \right) }{\delta \left( -\delta + \sqrt{-8\alpha \delta + \delta ^2} \right) } \,\,\, \ne \,\,\, 0 \\ \\ \dfrac{\textrm{d}}{\textrm{d} \beta } \Big (\textrm{Re} \,\, \lambda _{2}(\beta ) \Big ) \Big |_{\beta \,=\, \beta _2}= & \dfrac{\lambda ^2 \left( -\sqrt{-8\alpha \delta + \delta ^2} + 8\alpha -\delta \right) }{\delta \left( \delta + \sqrt{-8\alpha \delta + \delta ^2} \right) ^2} \,\,\, \ne \,\,\, 0. \end{aligned}$$

Hence, *P* undergoes a supercritical *Hopf* bifurcation at $\beta = \beta _1$ and undergoes a subcritical *Hopf* bifurcation at $\beta = \beta _2$. $\square$

**Fig. 5:**
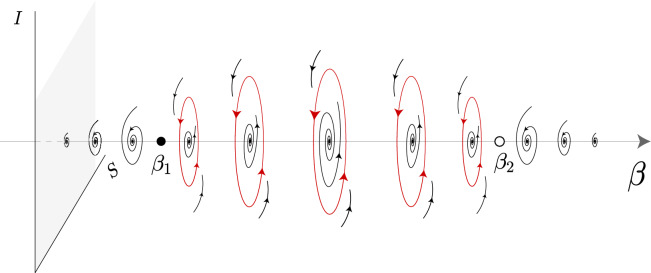
Schematic representation of Theorem 1 and Proposition 1 in the phase space (*S*, *I*) for different values of $\beta$. *P* is stabe for $\beta < \beta _1$ and $\beta> \beta _2$, and unstable for $\beta \in (\beta _1, \beta _2)$. The supercritical and subcritical *Hopf* bifurcations occur at $\beta = \beta _1$ and $\beta = \beta _2$, respectively. The attracting periodic solution is depicted in red.

As $\beta$ increases, the periodic solution appears for $\beta> \beta _1$ (supercritical *Hopf* bifurcation) and vanishes for $\beta> \beta _2$ (subcritical *Hopf* bifurcation). See the illustrations in Figs. 3 and 5, and the numerical simulation in Fig. 4.

**Fig. 6:**
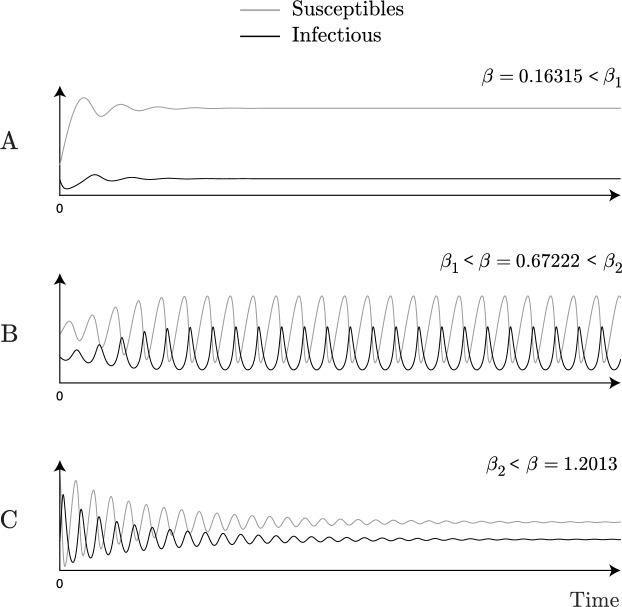
Numerical simulation for *S* and *I* over time $t \in \mathbb {R}_0^{+}$. *S* and *I* converge for $\beta < \beta _1$ and $\beta> \beta _2$, (**A** and **C)**, respectively, and are periodic in (**B)** for $\beta \in (\beta _1, \beta _2)$. Standard parameter values: $\lambda = 0.9$, $\alpha = 0.1$ and $\delta = 1.1$.

Figure 6 provides a numerical simulation of *S*(*t*) and *I*(*t*), $t \in \mathbb {R}_0^+$, for $\beta < \beta _1$, $\beta \in (\beta _1, \beta _2)$ and $\beta> \beta _2$, indicating that these are periodic for $\beta \in (\beta _1, \beta _2)$ and converge for the other ones. Theorem 1 is proved.

## Seasonality and chaotic dynamics: second main result

Considering seasonality in mathematical models of epidemiology makes systems richer and more complex to analyze. Some authors believe that including seasonality in dynamical systems makes the models more realistic. Another factor that contributes to the complexity of dynamical systems is the existence of *strange attractors* (observable chaos).

### Second main result

Before we present the second result of the article, we give the definition of *strange attractor* in accordance with Ruelle & Takens^20^.

#### Definition 1

(^20,21^,*adapted)* A (Hénon-type) *strange attractor* of a two-dimensional dissipative diffeomorphism, defined on a Riemannian manifold, is a compact invariant set $\Omega$ with the following properties:

1. the set $\Omega$ equals the topological closure of the unstable manifold of a hyperbolic periodic point;
2. the basin of attraction of $\Omega$ contains a non-empty open set ($\Rightarrow$ it has positive Lebesgue measure);
3. there is a dense orbit in $\Omega$ with a positive Lyapunov exponent.

A vector field possesses a *strange attractor* if the first return map to a cross section does.

#### Theorem 2

For $\mathcal {U}_2\subset \Lambda$ of Proposition 1 and for $\omega$ sufficiently large ($\omega \gg 1$), the following inequality holds for system (3):

$$\begin{aligned} \displaystyle \liminf _{\varepsilon \, \rightarrow \, 0^+} \, \left( \frac{\text {Leb} \left\{ \gamma \in [\,0, \, \varepsilon \,]: \, {f}_\gamma \,\, \text {exhibits a strange attractor}\right\} }{\varepsilon } \right)> 0, \end{aligned}$$

where Leb denotes the one-dimensional Lebesgue measure.

#### Proof of Theorem 2

We use Wang and Young’s theory^22^ to prove the existence of abundant *strange attractors* in (3) (Theorem 2). Given our Proposition 1 and Theorems 1 and 2 of^22^, we make the following statements:

- $\omega \gg 1$ by hypothesis, *i.e.* $\omega$ governs the frequency of $\Psi$, which may be interpreted as a seasonal constraint;
- $\beta _{\gamma }(t) = \beta \left( 1 + \gamma \Psi (\omega t) \right)$ (from (2)) is the radial kick of (3);
- the non-autonomous periodic forcing of 3 is at least $C^3$ and has two nondegenerate critical points;
- $\mathcal {C}$, that emerges from (3), is attracting.

From^23^[Section "Absense of seasonality: First main result"], one knows that there exists $\varepsilon> 0$ such that for Lebesgue-almost all $\gamma \in [0,\varepsilon ]$, the non-wandering set associated to $f_\gamma$ (system (3)) has rank-one *strange attractors*. For $\gamma =0$, the chaotic flow is realized for points that belong to the basin of attraction of $\mathcal {C}$, that is the phase space (*S*, *I*) for $\beta \in (\beta _1, \beta _2)$, defined as $\mathcal {B(\mathcal {C})}$ (see Figs. 2,3,4**(ii)**).

##### From an attracting curve to a strange attractor

For system (3), when $\gamma = 0$, the dynamics regarding the appearance of an attracting periodic solution is clear. The equilibrium point *P* loses its stability when it undergoes a supercritical *Hopf* bifurcation at $\beta = \beta _1$, giving rise to a limit cycle which increases in diameter the further it moves away from *P*, which is now unstable. At $\beta = \beta _2$, *P* undergoes a subcritical *Hopf* bifurcation and the limit cycle no longer exists and *P* is stable again. When we do not neglect seasonality, $\gamma \ne 0$, the disease transmission rate is given by a non-autonomous periodic function $\beta _{\gamma }$ which represents the periodic force of (3). As such, when $\omega$ is sufficiently large and the forcing is of a suitable type, then a *strange attractor* emerges from the bifurcation, instead of a limit cycle $\mathcal {C}$.

##### Quotient space and torus $\mathcal {T}$

Let $(\lambda , \beta , \alpha , \delta ) \in \mathcal {U}_2$, $\gamma \in [0,\varepsilon ]$ and $\omega \in \mathbb {R}^+$. Then, we can extend system (3) to the three-dimensional system in $\mathbb {R}^2 \times \mathbb {S}^1$:

10 $$\begin{aligned} {\left\{ \begin{array}{ll} \begin{array}{lcl} \dot{S} & = & \lambda - \beta \left( 1 + \gamma \Psi (\theta ) \right) SI^2 - \alpha S\\ \dot{I} & = & \beta \left( 1 + \gamma \Psi (\theta ) \right) SI^2 + \alpha S - \delta I \\ \dot{\theta } & = & \omega , \end{array} \end{array}\right. } \end{aligned}$$

where $\mathbb {S}^1$ is a quotient space.

###### Lemma 5

For $\gamma = 0$, $\omega \in \mathbb {R}^+$ and $(\lambda , \beta , \alpha , \delta ) \in \mathcal {U}_2$, the flow of (10) exhibits an attracting 2-dimensional torus $\mathcal {T}$, which is normally hyperbolic.

###### Proof

The proof follows the same lines of^16^: If $(\lambda , \beta , \alpha , \delta ) \in \mathcal {U}_2$, then the dynamics of (3) exhibits an attracting periodic solution in the phase space (*S*, *I*) (See Proposition 1). If we add the phase component $\dot{\theta } = \omega$, $\omega \in \mathbb {R}^+$, then an attracting two-dimensional torus emerges, which is normally hyperbolic (Normal hyperbolicity results from the torus being an attractor^24^), persisting for $(\lambda , \beta , \alpha , \delta ) \in \mathcal {U}_2$ and $\gamma , \omega \in \mathbb {R}^{+}$. $\square$

**Fig. 7:**
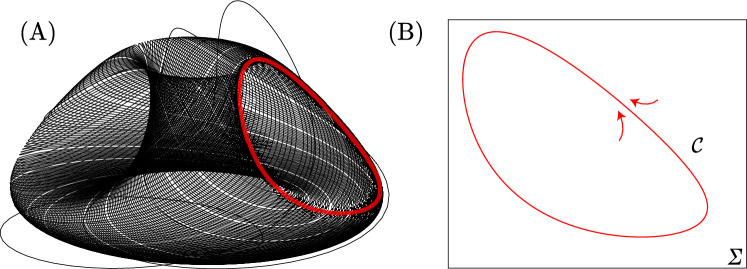
Attracting two-dimensional torus $\mathcal {T}$ for $\lambda = 0.9$, $\beta = (\beta _1 + \beta _2)/2$, $\alpha = 0.1$, $\delta = 1.1$, $\gamma = 0$ and $\omega = 0.1$. Illustration of $\mathcal {C}$ from a cross section $\Sigma$ to the torus $\mathcal {T}$ when $\gamma = 0$.

See Fig. 7 (A) to visualize the dynamics.

##### Torus-breakdown

Let $\mathcal {T}_{\gamma }$ be the hyperbolic continuation of the torus $\mathcal {T}$ and $\mathcal {J}_{(\gamma , \omega )}$ the first return map to $\Sigma$ (cross section to the torus) defined in $\left( \mathcal {B}(\mathcal {C}) \times \mathbb {S}^1 \right) \cap \Sigma$. For

1. $\boldsymbol{\gamma = 0}$ and $\boldsymbol{\omega \in \mathbb {R}^{+}}$ fixed: take a cross section $\Sigma$ to the torus $\mathcal {T}_0$ such that $\Sigma \cap \mathcal {T}_0$ is a smooth invariant curve $\mathcal {C}$ (Fig. 7 (B)). For $\omega \in \mathbb {R}^{+}$, at least one of the eigenvalues of $\textrm{d}\mathcal {J}_{(0,\omega )} \big |_{\mathcal {C}}$ has modulus less then 1;
2. $\boldsymbol{\gamma> 0}$ fixed and $\boldsymbol{\omega \in \mathbb {R}^{+}}$: $\mathcal {T}$ starts to desintegrate into a finite collection of periodic saddles and sinks, a phenomenon called *Torus-breakdown*;
3. $\boldsymbol{\gamma> 0}$ fixed and $\boldsymbol{\omega \gg 1}$: emergence of *strange attractors* created by stretch-and-fold type actions (*sustained chaos*)^22^.

**Fig. 8:**
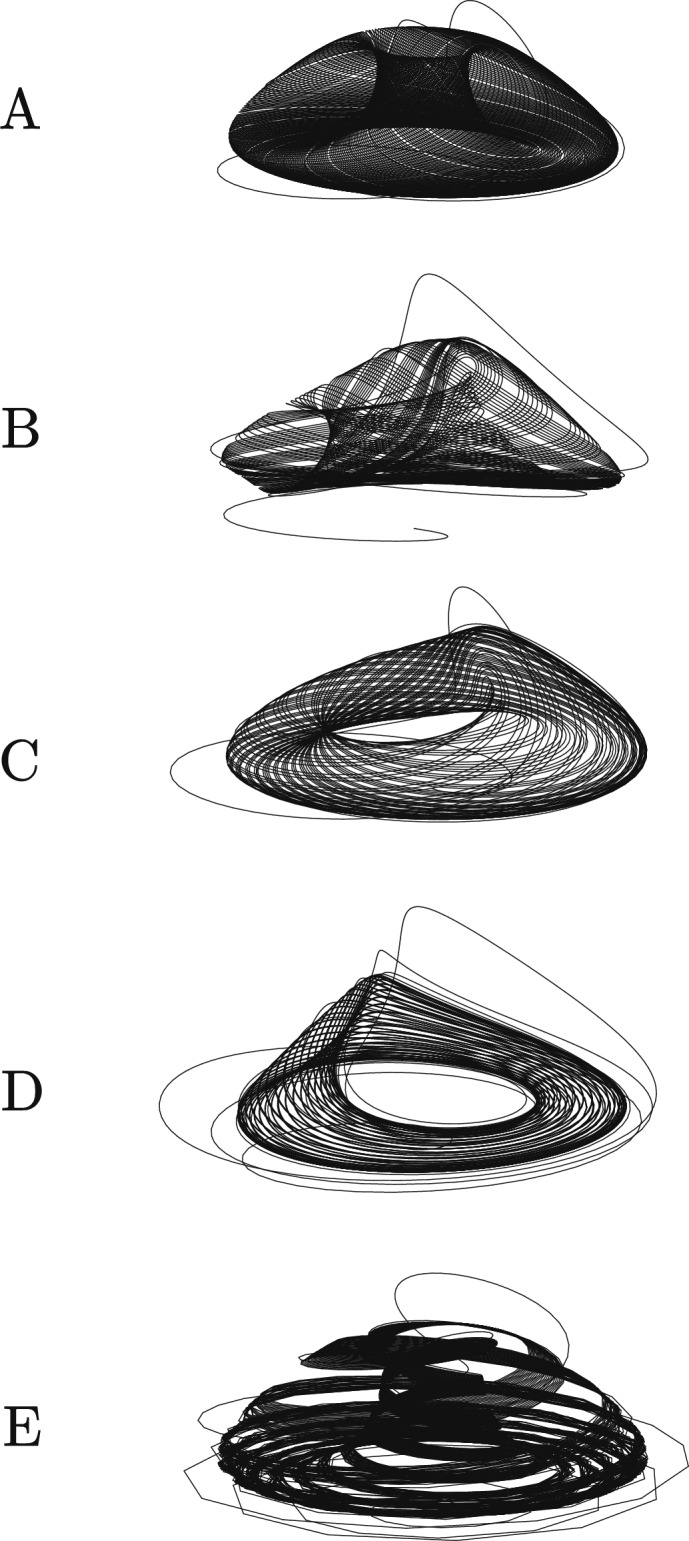
Simulation of a *Torus-breakdown*. (**A**) $\gamma = 0$, $\omega = 0.1$. (**B)** $\gamma = 0.001$, $\omega = 0.5$. (**C)** $\gamma = 0.001$, $\omega = 1$. (**D)** $\gamma = 0.001$, $\omega = 1.5$. (**E)** $\gamma = 0.001$, $\omega = 5$. Fixed parameter values: $\lambda = 0.9$, $\beta = (\beta _1 + \beta _2)/2$, $\alpha = 0.1$ and $\delta = 1.1.$.

The *Torus-breakdown* phenomenon is represented in Fig. 8. Theorem 2 is proved.

## Conclusion and discussion

In this paper we analyze a periodically-forced SIR model by including seasonality in the disease transmission rate $\beta$. The qualitative analysis of the model focused on flow dynamics with (10) and without (4) seasonality. In the absense of seasonality, we conclude that the endemic equilibrium point *P* unfolds into a supercritical *Hopf* bifurcation for values of $\beta = \beta _1$ and a subcritical *Hopf* bifurcation for values of $\beta = \beta _2$ (Theorem 1). If we consider seasonality in the disease transmission rate, then the dynamics exhibit *strange attractors* (observable chaos) in the neighborhood of $\beta = \beta _1$ and $\beta = \beta _2$ (Theorem 2).

### Biological interpretation of the results

The model we propose exhibits a unique equilibrium point, which is endemic (Lemma 2). In general, models derived from the classic SIR model have at least one disease-free equilibrium point (We have chosen to cite just a few references. Readers interested in more details and examples can consult the references we have provided.)^3,13,25^, where the second component of the equilibrium in the phase space (*S*, *I*) is zero. For this reason, it is impossible to eradicate the disease from the population in (1). However, by controlling the number of contacts between individuals, given by $\beta$, it is possible to control the stability of the endemic (Figs. 4, 5, 6). Although the endemic equilibrium point is unstable for a set of values of the disease transmission rate, a attracting periodic orbit appears and the flow is attracted to it, maintaining the stability of the endemic. Moreover, it would be expected that, for initial conditions of the form $(S_0, 0)$, the disease would eventually die out. Nevertheless, because the model incorporates infection via direct contact with the viral source $\alpha$, the disease persists within the population, as shown in Fig. 2.

The authors of^19^ analyze a similar model and found a supercritical *Hopf* bifurcation and a subcritical *Hopf* bifurcation. These dynamics allowed them to show the effects of diffusion on the outbreak. Our purpose is a little different: the double *Hopf* bifurcation gives a set of values of the disease transmission rate $\beta \in (\beta _1, \beta _2)$ for which we find an attracting periodic orbit $\mathcal {C}$. According to the theory of Wang and Young on Rank one *strange attractors*^22^, the existence of $\mathcal {C}$ is a necessary prerequisite for the existence of chaos in a periodically-forced systems. For intermediate values of the seasonal frequency, namely when $\omega$ is close to 1, the system exhibits a smooth deformation of the attracting torus before its complete breakdown. In this regime, the trajectories still display quasi-periodic behavior, but small distortions appear on the invariant torus due to the periodic forcing term $\gamma \Psi (\omega t)$. These distortions anticipate the transition to more complex dynamics observed for larger $\omega$, where the torus disintegrates into a collection of periodic saddles and sinks. This phenomenon marks the onset of the *torus-breakdown* process leading to the appearance of strange attractors, as illustrated in Fig. 8**E**. Therefore, despite the endemicity being controlled by the periodic attractor solution, when the system is subject to a high frequency of seasonality ($\omega \gg 1$), the probability of the emergence of *strange attractors* is very high (Theorem 2 and Fig. 8), making the dynamics unpredictable and the disease extremely difficult to control.

The analysis of the dynamics with chaotic behavior that arises from bifurcations offers important insights for understanding and managing epidemic outbreaks. It highlights how small changes in transmission parameters can induce large qualitative changes in the dynamics of the disease, thus helping policymakers formulate effective control measures. Nonetheless, the theoretical assumptions underlying the model represent an idealised view of reality and may ignore the biological and environmental variability present in real epidemic scenarios.

The numerical simulations provide a clear illustration and confirmation of the analytical conclusions. Figures 4, 5, 6, 7, 8 highlight the transition from periodic to chaotic regimes as the parameters $\beta _{\gamma }(t)$ and $\omega$ vary, confirming the theoretical predictions. From a biological perspective, these results emphasise how seasonality and contact intensity influence disease persistence. The numerical scheme adopted proved stable and efficient, ensuring reliable long-term integration of the system trajectories.

### Future work

This work motivates the development of a model that incorporates vaccination. However, the most suitable vaccination strategy has not yet been determined. In particular, it remains to be established whether a constant or a pulse vaccination scheme provides a better representation of the system’s behavior^13,26,27^. However, we conjecture that by introducing a new term into the model that maps the treatment (vaccination or other), we can get disease-free equilibria, as well as define regions where the disease does not persist in the population. Moreover, the emergence of a *Bautin* bifurcation in this model could be a reality and could be further investigated in future research.

## Data Availability

No datasets were generated or analysed during the current study. All results derive from analytical derivations and numerical simulations described in the article.
